# The association between transition into grandparenthood and Chinese older adults’ subjective well-being and health: a longitudinal study

**DOI:** 10.3389/fpubh.2025.1642496

**Published:** 2025-10-23

**Authors:** Huan Wang, Danyang Wang, Jiahao Yu, Jin Wang

**Affiliations:** ^1^Institute of Population Research, Hohai University, Nanjing, China; ^2^Nutrition Department, The First Affiliated Hospital with Nanjing Medical University, Nanjing, China

**Keywords:** grandparenthood transition, grandparental role, grandchild caregiving, well-being, health, older adults, China

## Abstract

**Background:**

Due to low birth rates, longer life expectancy, and later childbearing, an increasing number of individuals become grandparents later in life. The transition into grandparenthood—encompassing role acquisition, duration, and role engagement—is crucial for understanding the well-being and health of older adults. However, the connection between this transition and well-being and health outcomes among older adults remains underexplored, particularly in a Chinese context that emphasizes family lineage. This study addresses this gap by analyzing longitudinal data to examine how grandparenthood transitions relate to subjective well-being and health among Chinese older adults.

**Methods:**

Using longitudinal data from the China Longitudinal Aging Social Survey (CLASS) 2014–2020, this study investigates the associations between grandparenthood transition —specifically, grandparent role acquisition (i.e., becoming a grandparent), role duration (i.e., duration of being a grandparent), and role enactment (i.e., providing grandchild care)—and three well-being outcomes: life satisfaction, depressive symptoms, and self-rated health. Fixed-effects models are employed to account for unobservable time-invariant heterogeneity.

**Results:**

The transition into grandparenthood is associated with lower life satisfaction, increased depressive symptoms, and improved self-rated health among older adults in China, though these effects appear to be short-term. Additionally, the well-being and health benefits of becoming a grandparent are more pronounced for men compared to their female counterparts.

**Conclusion:**

The findings indicate that transitioning into the role of a grandparent, rather than caregiving per se, is negatively associated with certain aspects of well-being for older adults in the short term. However, this transition seems more beneficial for older men. These results underscore the relationship between becoming a grandparent and changes in the daily lives and well-being of older adults, suggesting that policymakers should develop targeted family support systems to help facilitate a positive adjustment during this transition, especially for grandmothers.

## 1 Introduction

China is experiencing one of the fastest demographic shifts worldwide. Since 2020, the total fertility rate has stayed between 1.2 and 1.3 births per woman, while life expectancy reached 77.93 years in 2022. The average age at first birth has also gone up, with Chinese women having their first child later, from 24.83 years in 2000 to 27.94 years in 2020 (1). As a result, more Chinese individuals are becoming grandparents later in life and are likely to spend a significant part of their old age in the grandparental role. Despite this growing trend, the transition into grandparenthood remains an underexplored area in aging research, especially in East Asia. Compared to the extensive literature on retirement, widowhood, and health shocks, studies focusing on grandparenthood are notably limited (2, 3).

Most existing research on grandparenthood has focused narrowly on grandchild care, treating it as the core expression of the grandparent role (4, 5). However, grandchild care can only occur when three conditions are met: (a) the acquisition of the role following the birth of a grandchild, (b) maintaining the status of being a grandparent over time (role duration), and (c) active engagement in caregiving behaviors (role enactment). Neglecting the acquisition and duration stages risks overlooking critical elements of how transition into grandparenthood influences older adults’ well-being and health. Prior studies from the U. S. have shown that the symbolic acquisition of a grandparental identity may itself affect psychological outcomes (6), and recent longitudinal research in Europe demonstrates that health trajectories can shift significantly at the moment of role transition (7).

A theoretical debate remains on whether becoming a grandparent enhances or harms one’s well-being. Role strain theory suggests that new caregiving expectations, financial transfers, and intergenerational negotiations can increase stress, especially in contexts lacking institutional support (8, 9). These burdens may fall disproportionately on older women, ethnic minorities, or individuals with fewer resources (10). In contrast, role enhancement theory posits that entering a new social role—particularly one tied to intergenerational connection—can enhance purpose, pride, and social integration, thus improving well-being (11). At the same time, patriarchal norms that concentrate caregiving expectations on older women may exacerbate gender disparities in psychological and physical outcomes (12). Meanwhile, empirical studies on the association between the transition to grandparenthood and the well-being and health of older adults remain limited. However, few have separated the different stages of grandparental role transition—acquisition, duration, and enactment—and most rely on cross-sectional data, making it difficult to identify causal effects and mainly focusing primarily on Europe and North America (2, 3, 10, 13, 14).

East Asian societies embrace family cultures that differ from those in the Western context, yet research on grandparenthood transitions from East Asian contexts remains limited (7). China offers an ideal setting for exploring this topic for several compelling reasons. On one hand, China faces the dual challenges of declining fertility rates and an aging population, meaning more older adults will have fewer chances to become grandparents, making the first transition especially important (15–17). On the other hand, Confucian ideals surrounding filial piety and lineage continuity create a unique cultural framework that heightens both the joy and obligation linked to grandparenthood, setting the Chinese experience apart from Western contexts where grandparenting is often more voluntary and less institutionalized (18).

Given the importance of grandparenthood transitions in China’s rapidly changing demographic landscape and the limited research on this topic in East Asian contexts, the present study draws on four waves of nationally representative data from the China Longitudinal Aging Social Survey (CLASS, 2014–2020). We employ fixed-effects models to examine the association between acquiring the grandparental role, the duration of this role, and its enactment, and the well-being and health among older adults in China. We further examine gender differences in these associations. By situating this transition within China’s unique cultural context and breaking down the process into key stages, this study helps develop a more nuanced understanding of how grandparenthood relates to well-being in later life and provides evidence to inform policies that support older adults in adapting to new family roles.

## 2 Literature review

### 2.1 Theoretical framework

The transition to grandparenthood is a significant life event that may be related to changes in the subjective well-being and health outcomes of older adults. Role enhancement and role strain theories provide valuable frameworks for understanding these effects (19, 20). According to role enhancement theory, becoming a grandparent can significantly boost older adults’ well-being by giving new meaning and purpose to their lives. Grandparenthood enables individuals to witness the continuation of their family lineage through their grandchildren, who share their genetic material (2). This role enables older adults to pass down traditions, values, and life experiences to younger generations, thereby reinforcing their family identity and maintaining their position as family authorities (21). These processes enhance psychological well-being by strengthening emotional bonds and support networks within the family (11).

Conversely, role strain theory suggests that becoming a grandparent can present significant challenges as older adults attempt to manage the demands of this new role (5, 19). These additional responsibilities may negatively affect their subjective well-being, particularly when they become overwhelming. Role overload occurs when individuals struggle to meet the demands of multiple roles due to limited time, energy, or financial resources (20, 22). For instance, research in Europe has shown that older adults often reduce their work hours after becoming grandparents in order to provide childcare, which can lead to lower income and increased psychological stress (23). However, traditional role theories primarily emphasize role performance, often overlooking the processual nature of role transitions, which include role acquisition and role duration.

### 2.2 Grandparenthood and older adults’ well-being and health

Extensive research has shown that major life transitions such as retirement, health decline, spousal loss, and changes in living arrangements greatly impact individuals’ well-being in later life (24–29). However, studies on the transition to grandparenthood are relatively limited in comparison.

The existing research on grandparenthood and its associated well-being and health outcomes among older adults has focused chiefly on grandchild care, yielding a complex and inconsistent pattern of findings. Several investigations suggest that moderate caregiving intensity may be associated with health improvements among grandparents. For example, Wang et al. (16) found that non-intensive grandparenting reduced depressive symptoms among Chinese grandparents, while Korean studies similarly demonstrated that moderate caregiving intensity was linked to better psychological well-being (30–33). Conversely, other research indicates that high-intensity caregiving may have detrimental effects. Brunello and Rocco (34) found that a 10-h monthly increase in European grandparental care could lead to depressive symptoms, while overly intensive caregiving often exceeds grandparents’ capacity and triggers role strain (35, 36). Furthermore, some studies report null associations between caregiving intensity and health outcomes. Danielsbacka et al. (37) employed fixed-effects longitudinal analyses and found that within-individual changes in caregiving intensity showed no causal associations with most health indicators, except for a modest association with activities of daily living.

These inconsistent findings may stem from several methodological, social-demographic and contextual factors. Methodological differences—including cross-sectional versus longitudinal designs, varying measures of grandparental involvement (from occasional babysitting to custodial care), and different well-being indicators—significantly affect study comparability and conclusions (25, 38). Besides, these studies often treat ‘grandparent’ as a fixed identity, lacking a process-oriented perspective on dynamic transformation. Additionally, individual and family circumstances, such as grandparents’ gender, age, and socioeconomic status, as well as the number and age of grandchildren being cared for, may further contribute to divergent conclusions (6, 6, 17, 39). Cultural and institutional contexts across countries, particularly whether welfare state provisions and social expectations frame grandparenthood as a choice or obligation, also influence its association with well-being (40).

The limited research specifically examining the transition to grandparenthood has also produced equally inconsistent findings. Several European studies have identified positive associations, with investigations reporting that becoming a grandparent significantly increases life satisfaction compared to individuals without grandchildren (37, 40). However, other studies have found negligible effects. For instance, a Finnish study reported no correlation between grandparenthood and self-rated happiness (41), while additional investigations revealed only weak associations with subjective well-being (13). Conversely, some evidence suggests negative associations. Leimer and colleagues discovered that becoming a grandparent generally lowers subjective well-being, though it does not significantly affect physical health or cognitive skills (38). Despite these empirical contributions, contemporary research continues to treat grandparenthood as a static condition rather than examining it as a dynamic life course transition. Studies rarely differentiate between the distinct phases of role acquisition, duration of involvement, and behavioral enactment of grandparenting responsibilities. Furthermore, the existing literature remains predominantly centered on Western contexts, with limited systematic exploration of diverse cultural settings such as China, where traditional Confucian values and deeply embedded intergenerational norms may fundamentally shape both the expectations and experiences of grandparenting.

### 2.3 Grandparenthood in the Chinese context and gender norms

China offers an ideal setting for studying grandparenthood due to its rapidly aging population and strong Confucian values that highlight family lineage continuation (42, 43). Traditional filial culture places great importance on the role of grandparents in older adults’ lives (44–46). Despite recent declines in fertility, Chinese older adults become grandparents earlier and hold this role longer than their Western counterparts, with over 80% becoming grandparents by age 55, compared to 70–80 years in Europe (17). However, due to delays in first marriages and the timing of first births among their adult children, more individuals are becoming grandparents later in life (1).

Gender significantly influences the experience of grandparenthood in China, reflecting deeply rooted cultural norms that assign different roles and expectations to men and women. In line with traditional gender-based divisions of labor, older women usually serve as primary caregivers for grandchildren, engaging in daily hands-on care, while older men often view grandchildren as representatives of generational continuity (47, 48).

Emerging research suggests substantial gender differences in the relationship between becoming a grandparent and the health and well-being of older adults. Luo et al. (49) found that grandparenting is negatively associated with depression for grandfathers but not for grandmothers, suggesting that men may derive greater psychological benefits from caregiving roles that traditionally fall outside their expected gender norms. Conversely, Wu et al. (7) revealed that transitioning to grandmotherhood was associated with increased risk of functional limitations, reflecting the physical toll of intensive caregiving that disproportionately falls on women. Tanskanen et al. (14) similarly found that transition to grandmotherhood increased life satisfaction more than transition to grandfatherhood, providing some support for evolutionary theories predicting stronger emotional connections between grandmothers and grandchildren. Despite increasing attention to gender differences in grandparenting, significant gaps remain in understanding how the transition to becoming a grandparent differently impacts men and women. Most existing research has focused on differences in caregiving practices rather than thoroughly examining how role acquisition, duration, and enactment might interact with gender to shape well-being and health outcomes.

### 2.4 The present study

To address the research gaps in the literature above, this study aims to explore the association between the first-time transition to grandparenthood and the well-being and health of older adults in the Chinese context. Specifically, based on the literature review, we hypothesize that the acquisition of the grandparental role is generally associated with improved well-being (H1), as supported by role enhancement theory and the traditional Chinese cultural emphasis on family continuity. However, in line with recent longitudinal findings, we anticipate that the effects of grandparenthood on well-being will vary over time, with more pronounced improvements during the immediate post-transition period, followed by a phase of adaptation and stabilization (H2). Furthermore, in line with traditional Chinese culture and the collectivist values surrounding caregiving, we hypothesize that providing grandchild care is associated with better well-being and health outcomes among older adults compared to not providing such care (H3). Finally, regarding gender norms related to the grandparent role, we suggest that female older adults may experience increased role pressure, leading to more negative well-being and health outcomes compared to their male counterparts (H4).

## 3 Methods

### 3.1 Data and sample

The data for this study were drawn from the China Longitudinal Aging Social Survey (CLASS, http://class.ruc.edu.cn/index.htm). This nationally representative survey focuses on community-dwelling older adults aged 60 and above, collecting information on demographic and socioeconomic status, retirement, health, and family dynamics. CLASS was initiated in 2014 using a stratified, complex multistage probability sampling method, with face-to-face interviews conducted biennially. For this study, data from 2014 (11,511 observations), 2016 (11,471 observations), 2018 (11,418 observations), and 2020 (11,398 observations) waves were utilized.

Since the transition into grandparenthood is an ongoing process, we only included respondents with living adult children who participated in at least two survey waves, resulting in the exclusion of 13,758 observations. To examine the effects of first-time transitions into grandparenthood on well-being, we excluded respondents who were already grandparents at their first interview (12,176 observations excluded) or who did not assume the grandparent role by the final interview during the study period (7,247 observations excluded), resulting in the removal of 19,423 observations. Considering that older adults with limited activities of daily living (ADL) functions and those aged 80 and above are less likely to provide grandchild care, we excluded these two groups (325 and 591 observations, respectively) to minimize estimation bias. Furthermore, cases with missing data on key outcome variables were removed (132 observations excluded). The final sample comprised 3,164 individuals, resulting in a total of 11,569 observations across the study period.

### 3.2 Variables

#### 3.2.1 Dependent variables

Subjective well-being in later life is commonly understood as a multidimensional self-assessment of overall life circumstances and emotional health, combining a cognitive-evaluative component (life satisfaction) with an affective component (positive feelings and lack of depressive symptoms) (50, 51). Health is similarly perceived as a general assessment of one’s physical condition, reliably predicting morbidity and mortality (52). Consistent with these definitions and established practices in grandparenthood research (6, 38, 53), we utilized three indicators to evaluate the subjective well-being and health of older respondents: life satisfaction, depressive symptoms, and self-rated health.

##### 3.2.1.1 Life satisfaction and self-rated health

Respondents were asked to assess the extent to which they were satisfied with their overall life and health status using a 5-point Likert scale ranging from 1 (*not at all satisfied/very poor*) to 5 (*completely satisfied/excellent*). In this study, life satisfaction and self-rated health were treated as continuous variables, with higher scores indicating higher life satisfaction and better health status. This approach was based on findings from previous studies, which indicated no significant differences in regression estimations between continuous and ordered coding procedures (17, 54).

##### 3.2.1.2 Depressive symptoms

Depressive symptoms were assessed using a revised version of the Center for Epidemiologic Studies Depression Scale (CES-D), which includes nine items and has been empirically validated among Chinese older adults (18, 55). Respondents reported the frequency of experiencing various depressive symptoms over the past week, covering three items for positive affect, two for negative affect, two for feelings of marginalization, and two for somatic symptoms. Responses were scored on a 3-point scale ranging from 1 (*rarely or none*) to 3 (*most or all the time*). The total CES-D9 score ranged from 9 to 27, with higher scores indicating more severe depressive symptoms.

#### 3.2.2 Independent variables: transition into grandparenthood

This study investigated the first-time transition into grandparenthood through three main aspects: (a) grandparental role acquisition, assessing whether respondents adopted the new identity of being grandparents; (b) grandparental role duration, measuring the time elapsed since respondents assumed their grandparental role; and (c) role enactment, evaluating whether participants provided care for their grandchildren.

##### 3.2.2.1 Grandparental role acquisition

To identify whether respondents transitioned into the grandparental role, we compared grandchild status reported by respondents across two successive waves. In each wave, the CLASS survey collected data on grandchildren by asking, “Does this [child’s name] have a child under 18?” Based on this information, we created a binary variable to indicate the attainment of grandparent status, defined as the increase in the total number of grandchildren from 0 to at least one across their adult children.

##### 3.2.2.2 Grandparental role duration

We constructed a continuous variable to measure the duration since respondents transitioned into the grandparental role. This variable was derived by combining information on the reported birth of a new grandchild and the interview year. Since the CLASS survey did not collect specific birth years for grandchildren, we identified whether a respondent became a grandparent between two consecutive waves. For the first observation where a respondent transitioned into grandparenthood, we assigned a duration of one year, reflecting the average interval between two survey waves. Subsequent years of observation were used to accumulate the duration of grandparenthood over time.

##### 3.2.2.3 Grandparental role enactment

To measure whether respondents provided care for their grandchildren, we utilized a binary variable based on their responses to the question, “Do you regularly provide care for a child under 18 years old belonging to [child’s name]?” However, it was not possible to distinguish care provided exclusively to newborn grandchildren from care commitments for other grandchildren. Additionally, this question was only included in the 2014, 2018, and 2020 survey waves.

#### 3.2.3 Control variables

We controlled a range of demographic, socioeconomic, family, and health variables to address potential confounders in the relationship between transition into grandparenthood and the well-being of older individuals.

Time-varying demographic and socioeconomic factors included age category (0 = under 70 years old; 1 = between 70 and 80 years old), marital status (0 = without a partner; 1 = with a partner), living arrangement (0 = living without adult children; 1 = living with adult children), working status (0 = unemployed; 1 = employed), residence region (0 = rural area; 1 = urban area), and pension enrollment (0 = without any pension; 1 = with at least one pension). Gender was treated as a time-invariant dummy variable (0 = female; 1 = male).

Family-related factors included bi-directional intergenerational support, encompassing financial, instrumental, and emotional support. Financial support was measured as the average financial or in-kind assistance exchanged between older respondents and their adult children, expressed in logarithmic form. Instrumental support was evaluated using a five-point scale, ranging from “hardly ever” (= 0) to “almost every day” (= 4). At the same time, emotional closeness was assessed as the average emotional closeness score reported for each adult child, ranging from “not close” (= 0) to “close” (= 2). The health selection effect in intergenerational caregiving remains a possibility, as healthier older adults are more likely to provide care. To reduce this bias, we adjusted for variables indicating the older adults’ health status. Health conditions were assessed using three dummy variables: instrumental activities of daily living (IADL; 0 = no limitations, 1 = some limitations), pain (0 = no pain, 1 = some pain), and chronic disease (0 = none, 1 = at least one chronic condition).

### 3.3 Analysis strategy

We first presented the descriptive statistical characteristics of the older adults at both the initial and final interviews. Subsequently, we estimated the well-being effects of the transition into grandparenthood using linear fixed-effect regression models. The fixed-effect model captures within-individual changes in older adults’ well-being associated with the transition into grandparenthood (56), specifically focusing on the shift from not having grandchildren to having at least one grandchild, the time duration since becoming a grandparent, and providing childcare. A key advantage of the fixed-effect regression model is its ability to control for unobserved time-invariant individual heterogeneity, such as personal traits or genetic predispositions, which could otherwise bias the results (12, 56). The general fixed-effect regression model can be described as follows (see Equation 1).


(1) $$Y_{\mathrm{it}}=\beta_{1}\times G_{\mathrm{it}}+\beta_{2}X_{\mathrm{it}}+\alpha_{i}+\mu_{\mathrm{it}}$$

where Y_it_ denotes the independent variable (i.e., life satisfaction, depressive symptoms, and self-rated health) in wave t; G_it_ indicates the grandparenthood variables, including becoming a grandparent, time since assuming a grandparent role, and grandchild care providing; $X_{\mathrm{it}}$ is a vector of control variables; $\alpha_{i}$ are individual-level fixed-effects; $\mu_{\mathrm{it}}$ is the error term.

We conducted separate regression analyses to explore different aspects of the grandparenthood transition and their specific relationships with older adults’ well-being and health. Instead of including all three grandparenthood variables in a single model, we estimated separate models for each to avoid potential multicollinearity and to facilitate more straightforward interpretation. First, we examined the association between becoming a grandparent by focusing on participants who were not grandparents at baseline and assessed whether this transition was related to changes in their well-being and health outcomes (Panel A). Second, we analyzed how the length of time since becoming a grandparent was associated with well-being and health, examining variations based on the time passed since assuming the role compared to before becoming a grandparent (Panel B). Third, we specifically analyzed grandparents to explore the relationship between providing childcare for grandchildren and their well-being, as this required focusing on a different sample—those who were already grandparents (Panel C). Finally, we tested whether gender moderated the relationship between all three grandparenthood variables and well-being and health outcomes within each panel.

In longitudinal data analysis, addressing endogeneity is crucial to avoid biased estimates. However, in this study, the transition into grandparenthood was not significantly influenced by endogenous factors related to the variables of interest. The decision to take on a grandparent role and the duration of this role are more likely determined by the choices of adult children rather than by the well-being and health of the grandparents themselves. Additionally, when examining the impact of providing childcare, individuals with limitations in daily activities were excluded from the analysis. Moreover, we accounted for chronic conditions, pain, and the ability to perform instrumental activities of daily living to minimize potential biases related to selective grandparenting. To assess the robustness of our fixed-effects estimates and validate our findings, we conducted additional analyses using alternative regression approaches. Specifically, we utilized pooled ordinary least squares (POLS) and random-effects (RE) regression models as sensitivity checks (57). While the fixed-effects model remains our preferred choice because it controls for unobserved time-invariant heterogeneity, these alternative models offer valid comparisons to examine the consistency of our results across different assumptions (58).

## 4 Results

### 4.1 Descriptive statistics

Table 1 presents the descriptive statistics for participants (*n* = 3,164) who transitioned into the grandparent role during the study, with a total of 11,569 person-years of observation. At the first interview, 72% of participants were older adults aged 70 years or below, and 47% were female. Most respondents were not living with their adult children (48%), resided in urban areas (66%), were married or cohabiting with a partner (78%), were employed (92%), and were enrolled in a pension scheme (75%). Regarding health status, only 17% of participants reported limitations in instrumental activities of daily living, while 59% had at least one chronic disease. Over the study period, participants transitioned into grandparenthood and, on average, assumed the role of a grandparent for 2.63 years. During this time, 44% of grandparents provided some form of care for their grandchildren. Before becoming grandparents, participants demonstrated moderate levels of health and well-being, as reflected in measures such as life satisfaction, depressive symptoms, and self-rated health. However, a slight decline in well-being and health was observed following the transition to grandparenthood.

**Table 1:** Descriptive statistics for the study sample at first/last interviews (*N* = 3,164).

Variables	First interview	Last interview
	Mean (SD)	Mean (SD)
Dependent variables		
Life satisfactory (range = 1–5)	3.87 (0.85)	3.77 (0.84)
Depressive symptoms (range = 9–27)	14.35 (3.43)	15.88 (3.29)
Self-rated health (range = 1–5)	3.43 (0.98)	3.46 (0.87)
Independent variables		
Grandparental role acquisition (ref = not yet)	n.a.	100
Grandparent role duration (range = 1–5)	n.a.	2.63 (1.54)
Grandparent role-playing (ref = not providing grandchild care)	n.a.	0.44
Moderators		
Gender (ref = female)	0.53	0.53
Controls		
Receiving financial support from adult children (natural log)	5.74 (3.08)	6.56 (2.43)
Providing financial support to adult children (natural log)	2.80 (3.51)	3.50 (3.52)
Receiving instrumental support from adult children (range = 0–4)	1.40 (1.60)	1.12 (1.41)
Providing instrumental support to adult children (range = 0–4)	1.68 (1.37)	2.07 (1.22)
Emotional closeness (range = 0–2)	1.86 (0.34)	1.85 (0.35)
Age category (ref = 60–70 years old)	0.28	0.44
Marital status (ref = without a partner)	0.78	0.79
Living arrangement (ref = living with adult children)	0.48	0.38
Urban region (ref = rural)	0.66	0.63
Working status (ref = unemployed)	0.92	0.95
Pension enrollment (ref = without any pension enrollment)	0.75	0.79
IADL (ref = no limit)	0.17	0.14
Chronic conditions (ref = none)	0.59	0.79
Pain suffering (ref = feeling no pain)	0.38	0.48

Data source: CLASS waves 2014, 2016, 2018, 2020; n.a. = not applicable; SD = standard deviation.

### 4.2 The well-being effects of transition into grandparenthood among Chinese older adults

Table 2 presents the fixed-effect model estimates examining the relationship between transitioning into grandparenthood and its association with the well-being and health of the study participants. Our findings reveal that becoming a grandparent is associated with a reduction in life satisfaction (𝛽 = − 0.179, *p* < 0.05; see column 1) and an increase in depressive symptoms (𝛽 = 0.793, 𝑝 < 0.001; see column 4) compared to the pre-transition phase. However, we observed a slight improvement in self-rated health following the transition to grandparenthood (𝛽 = 0.139, *p* < 0.001; see column 7).

**Table 2:** Abbreviated results for the association between transition into grandparenthood and well-being and health among Chinese older adults: fixed-effects models.

Variables	Life satisfaction			Depressive symptoms			Self-rated health		
	(1)	(2)	(3)	(4)	(5)	(6)	(7)	(8)	(9)
Panel A (*N* = 11, 569)									
Grandparental role acquisition (ref = not yet)	−0.179* (0.041)			0.793*** (0.177)			0.139*** (0.042)		
Controls	Yes			Yes			Yes		
Adjusted R^2^	0.065			0.168			0.132		
Panel B (*N* = 11, 569)									
Duration of grandparent role (ref = not yet)									
1 year		−0.074* (0.041)			0.741*** (0.177)			0.140*** (0.042)	
3 years		−0.157*** (0.059)			0.697*** (0.256)			0.121* (0.061)	
5 years		−0.276** (0.085)			−0.425 (0.363)			0.115 (0.087)	
Controls		Yes			Yes			Yes	
Adjusted R^2^		0.066			0.178			0.131	
Panel C (*N* = 5,007)									
Providing grandchild care (ref = not providing)			−0.037 (0.104)			0.009 (0.651)			0.269*** (0.090)
Controls			Yes			Yes			Yes
Adjusted R^2^			0.226			0.177			0.240

Data source: we utilized data from the 2014, 2016, 2018, and 2020 CLASS waves to estimate the acquisition and duration of the grandparental role. To estimate grandchild caregiving, we skipped the 2016 wave because related information was missing. All models adjust for category, urban region, marital status, living arrangement, employment status, pension enrollment, bi-directional intergenerational support, instrumental activities of daily living (IADL), pain and suffering, and chronic conditions. * *p* < 0.05, ** *p* < 0.01, *** *p* < 0.01. Robust standard errors are in parentheses.

Additionally, we explored variations in the effects of grandparenthood on well-being and health over time (see columns 2, 5, and 8). The estimates suggest an irregular temporal pattern in the effects of assuming the grandparent role on our main dependent variables. Specifically, life satisfaction experienced a moderate decline in the first year of becoming a grandparent (𝛽 = − 0.074, 𝑝 < 0.05), followed by a more substantial decline in the third year (𝛽 = − 0.157, 𝑝 < 0.001) and an even greater decrease in the fifth year (𝛽 = − 0.276, 𝑝 < 0.01), indicating a progressive reduction in life satisfaction over time. For depressive symptoms and self-rated health, our analysis identified a gradual change over one to three years. Depressive symptoms increased during this period (1 year: 𝛽 = 0.741, 𝑝 < 0.001; 3 years: 𝛽 = 0.697, 𝑝 < 0.001), while self-rated health showed improvements (1 year: 𝛽 = 0.140, 𝑝 < 0.001; 3 years: 𝛽 = 0.121, 𝑝 < 0.05). However, no long-term effects were observed for depressive symptoms or self-rated health beyond three years.

Finally, we examined the association between providing grandchild care and the well-being and health outcomes of older adults (see columns 3, 6, and 9). Among grandparents engaged in childcare, significant improvement was observed only in self-rated health (𝛽 = 0.269, 𝑝 < 0.001; see column 9). No significant associations were found between providing grandchild care and life satisfaction and depressive symptoms.

### 4.3 The moderating role of gender

Table 3 presents the empirical results examining the moderating effect of gender on the relationship between the transition into grandparenthood and the well-being and health of older adults in China. Our findings reveal a significant decrease in life satisfaction among Chinese older women upon assuming the role of a grandparent (𝛽 = − 0.177, 𝑝 < 0.001; see column 1). In contrast, a notable increase in life satisfaction is observed for Chinese older men (𝛽 = 0.185, 𝑝 < 0.01), indicating that the benefits of grandparenting are more pronounced for men than for women. However, no significant gender differences are found in the relationship between assuming a grandparent role and depressive symptoms or self-rated health (see columns 2 and 3).

**Table 3:** Abbreviated results for moderating effects of gender on the relationship between transition into grandparenthood and the well-being and health among Chinese older adults: fixed-effects models.

Variables	Life satisfaction	Depressive symptoms	Self-rated health
	(1)	(2)	(3)
Panel A (*N* = 11, 569)			
Grandparent role acquisition (ref = not yet)	−0.177*** (0.063)	0.955*** (0.254)	0.140** (0.062)
Grandparent role acquisition × gender (ref = female)	0.185** (0.077)	−0.298 (0.318)	−0.001 (0.079)
Panel B (*N* = 11, 569)			
Grandparent role duration (ref = not yet)			
1 year	−0.182*** (0.063)	0.878*** (0.258)	0.136** (0.062)
3 years	−0.215*** (0.080)	0.694** (0.342)	0.139* (0.082)
5 years	−0.411*** (0.131)	0.443 (0.562)	0.081 (0.128)
1 year × gender (ref = female)	0.203** (0.080)	−0.246 (0.333)	0.007 (0.081)
3 years × gender	0.105 (0.093)	0.020 (0.398)	−0.032 (0.095)
5 years × gender	0.392*** (0.144)	−1.405** (0.610)	0.055 (0.148)
Panel C (*N* = 5,007)			
Grandchild role enactment (ref = not providing grandchild care)	−0.148 (0.204)	−0.016 (0.798)	0.254 (0.176)
Providing grandchild care × gender (ref = female)	0.229 (0.345)	0.070 (1.451)	0.034 (0.234)

Data source: We utilized data from the 2014, 2016, 2018, and 2020 CLASS waves to estimate the acquisition and duration of the grandparental role. To estimate grandchild caregiving, we skipped the 2016 wave because related information was missing. All models adjust for age category, urban region, marital status, living arrangement, employment status, pension enrollment, bi-directional intergenerational support, instrumental activities of daily living (IADL), pain and suffering, and chronic conditions. * *p* < 0.05, ** *p* < 0.01, *** *p* < 0.01. Robust standard errors are in parentheses.

Our estimates also identify gender differences in the temporal effects of becoming grandparents. In the short term, transitioning into grandparenthood is associated with higher life satisfaction for grandfathers (1 year: 𝛽 = 0.203, 𝑝 < 0.01). Over a five-year period, this role transition has an even more pronounced positive outcome on life satisfaction (𝛽 = 0.392, 𝑝 < 0.001) and reduced depressive symptoms (𝛽 = − 1.405, 𝑝 < 0.01) for grandfathers compared to grandmothers. However, no gender differences are observed in both the short-term and long-term outcomes of transitioning into the grandparent role on self-rated health. Finally, our analysis finds no evidence of gender disparities in the association between providing grandchild care and the three well-being and health indicators examined in this study.

The results from these robustness checks generally support our main findings, although some estimated coefficients from the POLS and RE models are significantly larger than those obtained from the fixed-effects specifications (see Supplementary Tables S1–S4). This pattern indicates that unobserved individual characteristics may bias estimates in cross-sectional and random-effects analyses. The fixed-effects approach effectively addresses this potential bias by controlling for time-invariant unobserved factors, offering more conservative and reliable estimates. The convergence of directional effects across all three modeling methods reinforces confidence in our core findings. At the same time, the differences in magnitude highlight the importance of accounting for individual heterogeneity in life course transition studies.

## 5 Discussion

The transition into grandparenthood is a significant milestone in later life that is associated with the well-being and health of older adults in both positive and negative ways and therefore warrants further exploration. Drawing on longitudinal data from the China Longitudinal Aging Social Survey (CLASS, 2014–2020) and employing fixed-effects models, we examine the within-individual effects of becoming a grandparent on the well-being and health of older adults in China. We investigate key dimensions of the transition—including grandparental-role acquisition, role duration, and role enactment—as well as the moderating effect of gender. Contrary to prevailing assumptions, our findings suggest that the transition to grandparenthood, although deeply rooted in traditional Chinese norms, does not uniformly enhance the well-being and health of older adults, particularly in the short term.

First, our longitudinal analysis reveals that the transition into grandparenthood is associated with lower reported life satisfaction and increased depressive symptoms, offering limited support for Hypothesis 1. These findings align with role strain theory (19) and are consistent with previous studies conducted in Western contexts (6, 14, 37, 40). The adoption of a new grandparental role, accompanied by its associated responsibilities, may disrupt the equilibrium of older adults’ existing multiple roles, leading to psychological strain, stress accumulation, and emotional exhaustion (9). However, these findings do not imply a complete absence of positive effects associated with becoming a grandparent. In line with Chinese cultural norms that emphasize filial piety and intergenerational continuity, assuming the role of a grandparent often allows older adults to derive joy and fulfillment from the arrival of a new generation. This role offers a sense of purpose and value tied to extending the family lineage, contributing to their perception of life’s broader significance and their overall well-being (59–61). As such, while becoming a grandparent is associated with certain psychological rewards, our results suggest that the immediate emotional and psychological costs of assuming this new identity often outweigh these benefits.

However, our findings diverge from those of Wu et al. (7), who found that the transition into grandparenthood was associated with higher life satisfaction. This inconsistency may stem from differences in sample selection: their study focused on middle-aged and older adults aged 45 and above, while ours concentrates on a comparatively older cohort. These differences suggest that older individuals may face more pronounced role burdens during the transition into grandparenthood. In contrast, younger cohorts may be more inclined to interpret the new role as an opportunity to strengthen life purpose and emotional experience, thereby achieving a positive transformation in which role enhancement outweighs role strain.

In addition, we found that the adverse effects of transitioning into the grandparental role persist over an extended period, offering some evidence for Hypothesis 2. Our longitudinal estimations indicate that becoming a grandparent significantly lowers self-rated life satisfaction over time. However, these adverse effects diminish in the short term for depressive symptoms. Over recent decades, the transition into grandparenthood in China has been accompanied by shifts in family dynamics and resource allocation. Family support networks often redirect their attention toward newborns, which may inadvertently marginalize grandparents and contribute to a sustained decline in their life satisfaction (23, 47, 62–64). Interestingly, we observed that the transient negative effects on depressive symptoms disappear roughly three years after the transition. As younger generations grow up and grandparents acclimate to their new roles, they appear to adapt to the changed family dynamics (38, 40, 65–67). Specifically, once grandchildren reach preschool age (approximately three years old), grandparents often regain elements of their prior routines, alleviating the pressures and dilemmas associated with this role. Our findings are quite different from those of Leimer and van Ewijk, who drew data from ten Western European countries and found no clear pattern in the temporal effects of grandparental status on life quality or health, whether in the short or long term (38). One possible explanation is that our study, conducted within a culturally homogeneous Chinese context, helps reduce potential confounding caused by cultural differences.

Our study also highlights a positive relationship between transitioning into the grandparental role and self-rated health, providing some evidence in support of Hypothesis 1. These findings align with role-enhancement theory (20), which posits that adopting a new role can strengthen family support networks. Enhanced interaction between older adults and their adult children fosters a sense of belonging and promotes overall health (2, 59). Furthermore, the physical activities associated with caregiving may contribute positively to physical health, consistent with prior research (22, 56, 65). However, this beneficial effect on self-rated health may not persist in the long term, as the physical and emotional demands of grandparenting can become burdensome for some older individuals, potentially leading to stress and adverse health outcomes. Taken together, these findings illustrate the complex interplay of costs and rewards that accompany a new grandparental identity (61).

These findings reveal a significant difference in how grandparenthood is linked to self-rated health compared to subjective well-being measures among Chinese older adults. The improvement in self-rated health stems from the immediate strengthening of social support networks and enhanced life meaning (42), particularly within the Chinese cultural context, where grandchildren provide symbolic significance for older adults’ health (7). In contrast, psychological well-being indicators are more sensitive to the stress and challenges associated with transitioning into the grandparent role. While the grandparent role brings cultural satisfaction (46, 68), the accompanying responsibility expectations and caregiving burdens have a more direct association with psychological adaptation. Our findings demonstrate that positive health effects manifest immediately upon role acquisition, whereas psychological adaptation requires a longer period. Depressive symptoms improve after three years, while life satisfaction effects persist longer, suggesting that psychological role integration is more complex and enduring than physical health perception adjustments.

However, when caregiving is explicitly considered, we notice a different pattern in the link between the transition to grandparenthood and older adults’ well-being and health. Our analysis reveals significant associations between grandchild care and self-rated health, but not with life satisfaction or depressive symptoms. Therefore, we have limited evidence for hypothesis 3. This finding may reflect China’s deeply rooted caregiving culture, where grandparents see childcare as a routine family duty rather than a voluntary activity (69, 70). These findings remarkably contrast with prior research that focuses solely on the practice of the grandparent role (49, 59), which primarily highlights the positive effects of caregiving on individual health and well-being. Our results underscore the importance of a process-oriented perspective—treating caregiving as one component of the broader transition into grandparenthood. This approach enables a more nuanced understanding of the dynamic relationship between role transformation and older adults’ well-being.

Our study also reveals gender disparities in the effect of transitioning into grandparenthood on the well-being of older adults, with grandfathers experiencing more pronounced psychological benefits. This finding offers some support for Hypothesis 4, which concerns gender differences. Specifically, grandfathers report higher life satisfaction than grandmothers, and this gap widens over time. One plausible explanation is the emphasis on patrilineal succession in many male-dominated households, where men are more likely to prioritize lineage continuity and thus derive greater fulfilment from their grandchildren (47, 67). However, our analysis detects no significant gender difference in the associations between caregiving and subjective well-being and health across all three indicators—a pattern consistent with earlier studies (71, 72). One explanation is the directional consistency of grandchild caregiving’s linkage on grandparents, irrespective of gender (73). Another one relates to the specific nature of caregiving activities—playing, reading, or offering daily assistance—which have been shown to enhance older adults’ well-being (74, 75). Such activities may mitigate gender disparities because they are generally viewed as meaningful and rewarding by both grandmothers and grandfathers. However, our gender pattern contrasts with some European evidence: Tanskanen et al. (14) report that entry into grandmotherhood—but not grandfatherhood—raises life satisfaction in 13 European countries. This reversal suggests that cultural scripts may assign different expectations to grandmothers across contexts: in Europe, grandmothers may experience role enhancement under manageable caregiving demands, whereas in China, the heavier caregiving load may erode psychological well-being.

## 6 Conclusion

In traditional Chinese culture, family lineage and intergenerational continuity are deeply ingrained values that shape the social structure (54, 76). Despite declining fertility rates, these cultural expectations remain firmly entrenched (77). The transition into grandparenthood both embodies and reinforces these traditions. However, our findings reveal a complex relationship between cultural norms, grandparental roles, and well-being and health outcomes. The acquisition and duration of the grandparent role, rather than caregiving per se, are crucial elements linked to the well-being and health of older adults in China. While adherence to cultural expectations surrounding grandparenthood aligns with societal norms, it does not necessarily result in the anticipated health and well-being benefits for older adults. The grandparent role encompasses both positive dimensions, such as deriving satisfaction from continuing the family lineage, and challenges, including role conflicts and the pressures of responsibility. These dual facets create nuanced and sometimes contradictory effects on the well-being and health of older adults. Our study highlights the importance of examining these transitions through an individual systems framework that considers the intersection of cultural values, role adaptation, personal well-being, and related variations among subgroups. Future efforts should aim to balance the preservation of cultural traditions with the well-being and role adjustment of older adults as they navigate these transitions.

Our finding necessitates targeted interventions for older adults transitioning to grandparenthood in China. First, given decreased life satisfaction and increased depressive symptoms associated with becoming a grandparent, policymakers should establish mental health support programs including counseling, peer support groups, and stress management workshops for newly transitioned grandparents, with healthcare providers trained to address grandparenthood-related psychological challenges. Second, observed gender disparities necessitate differentiated community programs that cater to varying gender-specific needs, while also acknowledging traditional role expectations. Third, since negative effects diminish approximately three years post-transition, early intervention should target the immediate post-transition period when psychological strain peaks, facilitating gradual role adjustment through flexible caregiving arrangements. Finally, policies must balance traditional values of filial piety with protecting older adults’ mental health through promoting open family communication about caregiving expectations, developing respite care services, and incorporating regular well-being assessments in healthcare systems to maximize grandparenthood’s positive aspects while mitigating adverse effects.

This study has several limitations. First, our measurement of the transition to grandparenthood is constrained by the available data. Because the China Longitudinal Aging Social Survey (CLASS) does not record the exact birth dates of grandchildren, we cannot determine the precise timing of the transition or the length of time spent as grandparents. In addition, the CLASS data lack detailed information on caregiving responsibilities and the characteristics and circumstances of grandchildren, which limits our ability to comprehensively examine caregiving behaviors after the transition. Second, the data does not capture grandparents’ subjective attitudes toward their roles and caregiving responsibilities—specifically, whether these roles are undertaken voluntarily or imposed, which further restricts our understanding of the nuanced experience of grandparenthood. Third, regarding caregiving intensity, while our sample selection strategy of focusing on grandparents with grandchildren of similar ages helps control for potential variations in caregiving demands, the relatively consistent caregiving intensity across our sample also means there is insufficient variation to fully capture the differential effects of caregiving intensity in our fixed-effects models. Future longitudinal studies with more detailed caregiving measures and longer observation periods would be valuable for exploring the mechanisms through which different levels of caregiving intensity affect older adults’ subjective well-being.

Moreover, although our fixed-effects models account for unobserved heterogeneity, we cannot entirely rule out the possibility of endogeneity bias. Therefore, the results should be interpreted with caution regarding causality. Additionally, while this study quantitatively elucidates the complex relationship between the transition into grandparenthood and the well-being and health of Chinese older adults, as well as gender norm differences, further clarification of the underlying mechanisms through qualitative research is needed to provide policymakers with more targeted insights. Additionally, this study focuses exclusively on the association between becoming a first-time grandparent and the health and well-being of older individuals. Because older adults may have multiple children and, in light of China’s evolving reproductive policies, may become grandparents more than once, further research is needed to investigate the cumulative effects of overlapping grandparent roles on their health and well-being. Finally, our analysis does not fully account for other major life changes that may happen at the same time as becoming a grandparent, such as stopping work, health shock, or spouse loss, since only 0.3, 3.6, and 0.6% of the sample experienced such negative life events in the past 12 months. Nevertheless, the combined effects of multiple life transitions on the well-being of older adults deserve further research.

## Data Availability

Publicly available datasets were analyzed in this study. This data can be found at: http://class.ruc.edu.cn/index.htm.
