# Supplementary material for: The association between transition into grandparenthood and Chinese older adults’ subjective well-being and health: a longitudinal study
Source: Front Public Health. 2025 Oct 23;13:1642496. doi: 10.3389/fpubh.2025.1642496 (PMC12589045; doi:10.3389/fpubh.2025.1642496)
Supplement: Supplementary file 1 [file Data_Sheet_1.docx]

**Appendix**

Table A1. Association between Transition into Grandparenthood and Well-being among Chinese Older Adults: Abbreviated POLS Model Results

|  | Life satisfaction | | | Depression symptoms | | | Self-rated health | | |
| --- | --- | --- | --- | --- | --- | --- | --- | --- | --- |
|  | (1) | (2) | (3) | (4) | (5) | (6) | (7) | (8) | (9) |
| **Panel A** |  |  |  |  |  |  |  |  |  |
| **Grandparent role acquisition** (ref = not yet) | -0.116^***^ |  |  | 0.716*** |  |  | 0.100** |  |  |
|  | (0.043) |  |  | (0.165) |  |  | (0.043) |  |  |
| Controls | Yes |  |  | Yes |  |  | Yes |  |  |
| Constant | 3.252*** |  |  | 18.610*** |  |  | 3.395*** |  |  |
|  | (0.182) |  |  | (0.585) |  |  | (0.156) |  |  |
| Adjusted R^2^ | 0.056 |  |  | 0.136 |  |  | 0.180 |  |  |
| **Panel B** |  |  |  |  |  |  |  |  |  |
| **Duration of grandparent role assumption** (ref = not yet) |  |  |  |  |  |  |  |  |  |
| 1 year |  | -0.116^***^ |  |  | 0.716*** |  |  | 0.100** |  |
|  |  | (0.043) |  |  | (0.165) |  |  | (0.043) |  |
| 3 years |  | -0.116^***^ |  |  | 0.716*** |  |  | 0.100** |  |
|  |  | (0.043) |  |  | (0.165) |  |  | (0.043) |  |
| 5 years |  | -0.116^***^ |  |  | 0.716*** |  |  | 0.100** |  |
|  |  | (0.043) |  |  | (0.165) |  |  | (0.043) |  |
| Controls |  | Yes |  |  | Yes |  |  | Yes |  |
| Constant |  | 3.265*** |  |  | 18.674*** |  |  | 3.404*** |  |
|  |  | (0.183) |  |  | (0.493) |  |  | (0.156) |  |
| Adjusted R^2^ |  | 0.057 |  |  | 0.146 |  |  | 0.180 |  |
| **Panel C** |  |  |  |  |  |  |  |  |  |
| **Providing grandchild care** (ref = not providing) |  |  | 0.108* |  |  | -0.119 |  |  | -0.053 |
|  |  |  | (0.064) |  |  | (0.244) |  |  | (0.061) |
| Controls |  |  | Yes |  |  | Yes |  |  | Yes |
| Constant |  |  | 3.077*** |  |  | 18.425*** |  |  | 3.309*** |
|  |  |  | (0.312) |  |  | (0.882) |  |  | (0.252) |
| Adjusted R^2^ |  |  | 0.070 |  |  | 0.097 |  |  | 0.172 |

**Data source:** We used 2014, 2016, 2018, and 2020 wave data to estimate the time since becoming a grandparent and the time since the transition into grandparenthood; we also used 2014, 2018, and 2020 wave data for grandparenting.

**Note:** All models control for age category, residence region, marriage status, living arrangement, working status, pension enrollment, bi-directional intergenerational supports, IADL, pain suffering, and chronic conditions. * p<0.10, ** p<0.05, *** p<0.01. Robust standard errors are in parentheses.

Table A2. Gender Moderation in the Association between Transition into Grandparenthood and Older Adults' Well-being: Abbreviated POLS Model Results

|  | Life satisfaction | Depression symptoms | Self-rated health |
| --- | --- | --- | --- |
|  | (1) | (2) | (3) |
| Panel A |  |  |  |
| **Grandparent role acquisition** (ref= not yet) | -0.155*** | 0.893*** | 0.096* |
|  | (0.056) | (0.224) | (0.057) |
| Becoming a grandparent × gender (ref = female) | 0.082 | -0.325 | 0.002 |
|  | (0.074) | (0.289) | (0.076) |
| **Panel B** |  |  |  |
| **Duration of grandparent role assumption** (ref = not yet) |  |  |  |
| 1 year | -0.131** | 0.973*** | 0.114** |
|  | (0.057) | (0.230) | (0.058) |
| 3 year | -0.156** | 0.899*** | 0.067 |
|  | (0.070) | (0.275) | (0.072) |
| 5 year | -0.357*** | -0.057 | 0.038 |
|  | (0.110) | (0.512) | (0.107) |
| 1 year × gender (ref=female) | 0.084 | -0.311 | 0.004 |
|  | (0.077) | (0.307) | (0.078) |
| 3 years × gender | -0.013 | -0.066 | -0.001 |
|  | (0.091) | (0.349) | (0.091) |
| 5 years × gender | 0.381*** | -0.781 | 0.023 |
|  | (0.138) | (0.617) | (0.136) |
| **Panel C** |  |  |  |
| **Duration of grandparent role assumption** (ref = not yet) | 0.113 | 0.108 | -0.076 |
|  | (0.091) | (0.338) | (0.088) |
| Providing care × gender | -0.016 | -0.435 | 0.048 |
|  | (0.126) | (0.446) | (0.118) |

**Data source:** We used 2014, 2016, 2018, and 2020 wave data to estimate the time since becoming a grandparent and the time since the transition into grandparenthood; we also used 2014, 2018, and 2020 wave data for grandparenting.

**Note:** All models control for age category, residence region, marriage status, living arrangement, working status, pension enrollment, bi-directional intergenerational supports, IADL, pain suffering, and chronic conditions. * p<0.10, ** p<0.05, *** p<0.01. Robust standard errors are in parentheses.

Table A3. Association between Transition into Grandparenthood and Well-being among Chinese Older Adults: Abbreviated Random-Effects Model Results

|  | Life satisfaction | | | Depression symptoms | | | Self-rated health | | |
| --- | --- | --- | --- | --- | --- | --- | --- | --- | --- |
|  | (1) | (2) | (3) | (4) | (5) | (6) | (7) | (8) | (9) |
| **Panel A** |  |  |  |  |  |  |  |  |  |
| Grandparental role acquisition (ref = not yet) | -0.107** |  |  | 0.747*** |  |  | 0.117*** |  |  |
|  | (0.042) |  |  | (0.162) |  |  | (0.042) |  |  |
| Controls | Yes |  |  | Yes |  |  | Yes |  |  |
| Constant | 3.250*** |  |  | 18.684*** |  |  | 3.454*** |  |  |
|  | (0.169) |  |  | (0.561) |  |  | (0.147) |  |  |
| Adjusted R^2^ | 0.061 |  |  | 0.142 |  |  | 0.184 |  |  |
| **Panel B** |  |  |  |  |  |  |  |  |  |
| Duration of grandparent role assumption (ref = not yet) |  |  |  |  |  |  |  |  |  |
| 1 year |  | -0.085** |  |  | 0.792*** |  |  | 0.126*** |  |
|  |  | (0.042) |  |  | (0.166) |  |  | (0.042) |  |
| 3 years |  | -0.172*** |  |  | 0.829*** |  |  | 0.090* |  |
|  |  | (0.052) |  |  | (0.201) |  |  | (0.052) |  |
| 5 years |  | -0.171** |  |  | -0.438 |  |  | 0.080 |  |
|  |  | (0.073) |  |  | (0.321) |  |  | (0.072) |  |
| Controls |  | Yes |  |  | Yes |  |  | Yes |  |
| Constant |  | 3.273*** |  |  | 18.768*** |  |  | 3.464*** |  |
|  |  | (0.170) |  |  | (0.561) |  |  | (0.148) |  |
| Adjusted R^2^ |  | 0.062 |  |  | 0.152 |  |  | 0.185 |  |
|  |  |  |  |  |  |  |  |  |  |
| **Panel C** |  |  |  |  |  |  |  |  |  |
| Providing grandchild care (ref = not providing) |  |  | 0.033 |  |  | -0.131 |  |  | 0.046 |
|  |  |  | (0.068) |  |  | (0.245) |  |  | (0.059) |
| Controls |  |  | Yes |  |  | Yes |  |  | Yes |
| Constant |  |  | 3.211*** |  |  | 18.554*** |  |  | 3.232*** |
|  |  |  | (0.277) |  |  | (0.853) |  |  | (0.249) |
| Adjusted R^2^ |  |  | 0.074 |  |  | 0.111 |  |  | 0.174 |

**Data source:** We used 2014, 2016, 2018, and 2020 wave data to estimate the time since becoming a grandparent and the time since the transition into grandparenthood; we also used 2014, 2018, and 2020 wave data for grandparenting.

**Note:** All models control for age category, residence region, marriage status, living arrangement, working status, pension enrollment, bi-directional intergenerational supports, IADL, pain suffering, and chronic conditions. * p<0.10, ** p<0.05, *** p<0.01. Robust standard errors are in parentheses.

Table A4. Gender Moderation in the Association between Transition into Grandparenthood and Older Adults' Well-being: Abbreviated Random-Effects Model Results

|  | Life satisfaction | Depression symptoms | Self-rated health |
| --- | --- | --- | --- |
|  | (1) | (2) | (3) |
| **Panel A** |  |  |  |
| Grandparent role acquisition  (ref= not yet) | -0.163*** | 0.923*** | 0.113** |
|  | (0.056) | (0.219) | (0.057) |
| Grandparent role acquisition  × gender (ref = female) | 0.112 | -0.325 | 0.002 |
|  | (0.073) | (0.286) | (0.075) |
| **Panel B** |  |  |  |
| Duration of grandparent role assumption (ref = not yet) |  |  |  |
| 1 year | -0.146** | 0.951*** | 0.121** |
|  | (0.058) | (0.228) | (0.058) |
| 3 years | -0.182*** | 0.857*** | 0.092 |
|  | (0.069) | (0.271) | (0.071) |
| 5 years | -0.386*** | 0.182 | 0.057 |
|  | (0.110) | (0.490) | (0.107) |
| 1 year × gender (ref=female) | 0.120 | -0.299 | 0.008 |
|  | (0.077) | (0.306) | (0.078) |
| 3 years × gender | 0.030 | -0.042 | -0.011 |
|  | (0.088) | (0.347) | (0.089) |
| 5 years × gender | 0.377*** | -1.014* | 0.027 |
|  | (0.134) | (0.585) | (0.134) |
| **Panel C** |  |  |  |
| Providing grandchild care (ref = not providing) | 0.027 | 0.006 | 0.067 |
|  | (0.093) | (0.325) | (0.089) |
| Providing grandchild care × gender (ref=female) | 0.010 | -0.263 | -0.040 |
|  | (0.131) | (0.451) | (0.117) |

**Data source:** We used 2014, 2016, 2018, and 2020 wave data to estimate the time since becoming a grandparent and the time since the transition into grandparenthood; we also used 2014, 2018, and 2020 wave data for grandparenting.

**Note:** All models control for age category, residence region, marriage status, living arrangement, working status, pension enrollment, bi-directional intergenerational supports, IADL, pain suffering, and chronic conditions. * p<0.10, ** p<0.05, *** p<0.01. Robust standard errors are in parentheses
